# Sociodemographic Variables and Body Mass Index Associated with the Risk of Eating Disorders in Spanish University Students

**DOI:** 10.3390/ejihpe13030046

**Published:** 2023-03-14

**Authors:** María-Camino Escolar-Llamazares, María-Ángeles Martínez-Martín, María-Begoña Medina-Gómez, María-Yolanda González-Alonso, Elvira Mercado-Val, Fernando Lara-Ortega

**Affiliations:** 1 Department of Health Sciences, University of Burgos, Paseo Comendadores s/n, 09001 Burgos, Spain; 2 Department of Educational Sciences, University of Burgos, Avda, Villadiego, No.1., 09001 Burgos, Spain

**Keywords:** eating disorder, gender, age, university degree, body mass index, students

## Abstract

Background: The passage through university is a complex experience that can heighten personal susceptibility to eating disorders. The objective of this research is to determine how gender, age, course, educational faculty, and body mass index (BMI) can influence the risk of eating disorders among university students. Method: A transversal and descriptive study is conducted with a sample of 516 Spanish students (57.2% female, 42.8% male; *M_age_* = 21.7, *SD_age_* = 4.1) following 26 university degrees. The Inventory Eating Disorder-Reference criterion (EDI-3-RF) was administered to the students. Contingency tables were used between categorical variables with the chi-squared statistic, at a significance level of *p* < 0.05. The Student *t*-test was used for two independent samples and a one-way ANOVA test with the post hoc Bonferroni test for more than two groups. Pearson’s correlation and a simple linear regression analysis were used to analyze the relationship between the variables in its quantitative version. Results: It was found that the female students enrolled in the second year presented a greater obsession with thinness and body dissatisfaction (*p* = 0.029; *d* = 0.338); the male students practiced more physical exercise to control their weight (*p* = 0.003); and that students under the age of twenty (*p* < 0.010; *d* = 0.584) and students from both the Health (*p* = 0.0.13) and Law (*p* = 0.021) educational faculties showed greater bulimic behavior (*d* = 0.070). More females are underweight (*z* = 2.8), and more men are overweight (*z* = 2.4). Normal-weight students scored significantly higher in thinness obsession (*p* = 0.033). Overweight students scored significantly higher on thinness obsession (*p* < 0.001) and body dissatisfaction (*p* < 0.001). Obese students scored significantly higher on body dissatisfaction (*p* = 0.04). Conclusions: The data obtained in this study, reinforce the hypothesis that the female gender, at an age within the limits of early adolescence, in the first year of the degree courses, with specific university qualifications, and a high BMI constituted factors that could provoke an eating disorder. Consequently, it is necessary to implement preventive measures adapted to the circumstances of each university student.

## 1. Introduction

The passage through university for many young people represents a complex experience, in which they will encounter stressors that heighten their susceptibility to succumbing to mental health disorders of various kinds [1,2,3]. The scientific evidence suggests that both the particular characteristics of the life cycle and the demands of university life (heavier academic workload, adaptation to changing social solidarity and networks, greater autonomy, increased responsibility, vocational and academic failure, moving home, living alone) increase the probability of emergent emotional difficulties that, if not properly treated, will metamorphose into clinical disorders [1,4,5].

Eating disorders (ED) are hardly unknown to university students who are, in fact, a high-risk population, due in part to all the changes associated with the start of university life [6,7,8]. ED are serious mental disorders, related to body dissatisfaction, excessive preoccupation with food, weight, body image and what it represents. Likewise, they produce important behavioral alterations, as a consequence of the attempt to control the body and weight [9,10,11]. EDs represent a public health problem, given their prevalence, severity, prolonged clinical course, tendency to chronification, need for pluri- and interdisciplinary treatment, and recurrent hospitalization [5,12,13].

The scientific literature has established that over 60% of university students hold erroneous perceptions of their physical image, overestimating their body mass index (BMI) [14,15,16,17]. This body dissatisfaction accompanied by slimming diets constitutes one of the main risk factors of EDs [15,18,19,20,21,22]. Body dissatisfaction is characterized by the presence of value judgments about the body that do not match the actual characteristics [10,23,24]. Similarly, body dissatisfaction represents dissatisfaction with overall body shape and with the size of specific body parts (e.g., stomach, hips, thighs, and buttocks) that are of extraordinary concern to people with eating disorders [5,25,26].

Aspects such as feeling bloated after eating a normal amount of food are common features among people who feel dissatisfied with their own bodies [11,27,28]. Given that body dissatisfaction is endemic among young women in Western cultures it cannot be concluded that this construct alone causes EDs. However, it is a risk factor responsible for the initiation and maintenance of extreme weight control behaviors that lead to the development of EDs in those who are vulnerable [5,11,28].

On the other hand, weight assessment is important in EDs because it provides information about the context in which the symptoms occur. Moreover, it is necessary to understand subjective psychological distress related to figure and appearance [11,29,30]. Studies conducted on body dissatisfaction consistently show that it is positively correlated with body weight and body mass index (BMI). Similarly, BMI correlates positively with obsession with thinness and problematic eating patterns (binge eating) in response to negative emotional states. Finally, BMI is particularly important since a very low value of this index is a very serious sign of an eating disorder or physical illness [5,11,29].

In relation to gender, epidemiological studies have consistently made clear that an ED is more common among women than among men [15,18,22,31]. In this sense, Martínez-González, L. et al. [32] found a risk prevalence of 19% in a university population, which was higher among women. However, the impact of one ED or another has also been shown to affect the male population [15,31,33]. 

Although the masculine and feminine risk profiles for an ED differ, risk factors also arise due to peer pressures surrounding an ideal of beauty. In the case of men, an ideal musculature can never be an ideal of slimness. However, it also exposes men to ED risk behaviors, such as: the use of steroids, high consumption of laxatives, desire for greater muscular mass, dietary complements, diet and excessive sports activity [7,34,35,36].

Authors such as Sepúlveda et al. [37] and Chin et al. [10] signaled some significative gender-related differences in risk behavior. For example, vomiting was present in 9.6% of men as opposed to 16% of women and the use of laxatives to control body weight stood at 10.6% among men and 14.5% among women [38]. Other studies, such as the one by Lameiras et al. [39] with university students from the Autonomous Community of Galicia (Spain), affirmed that women showed higher levels of concern, due to weight and body image, and they resorted to diets to reach the ideal weight. Franco et al. [15] found that the three risk behaviors reported to a greater extent among women were compensatory behaviors, the use of slimming products, and binge eating, and among the men, compensatory behaviors, binge eating, and exercise to burn off calories.

In relation to age, González-Carrascosa et al. [7], Daly and Costigan [3] and Schilder et al. [40], among others, pointed out that the start of these disorders is habitually at prepubescent ages and early adolescence, with a higher percentage prevalence among young adults, who form the majority of the university population. An epidemiological study carried out by the *Asociación contra la Anorexia y la Bulimia* (ACAB) [Association against Anorexia and Bulimia] and Andersen [41] with universities (18–25 years old) from the Catalan Autonomous Community (Spain), confirmed that 11.48% presented a high risk of suffering from an ED, while 6.38% could have been suffering from it at that time. A significant proportion of university students therefore appear to be at-risk of developing an ED in the future. As much is also confirmed in the results of work completed at the Autonomous University of Madrid by Sepúlveda et al. [37] in which 14.9% of men and 20.8% of women presented a high risk of suffering an ED.

Although social pressures tend to be more intense at the adolescent stage, the cult of the body is maintained throughout all stages of life [42]. EDs are therefore increasingly present at early ages and are at the same time maintained at more advanced ages [7,18,43].

In studying the influence of the year in which students are enrolled, the scientific literature points to the importance of the first years of university. The first year is an especially critical period for the onset of pathological or disordered eating patterns. The increase in independence and responsibilities, as well as questions over personal identity all contributed in part to the pathological eating patterns [44,45].

Increased weight, by more than two-thirds, occurred among first-year students, during their first semester [46,47,48]. Body mass tended to be added, on average, by slightly over 1% [44,49]. Other studies have reported weight gain and increased body mass among students throughout the different university courses [44].

With regard to the selection of university qualification or degree, it could be influenced by pre-existing eating disorders. Personality traits, motivations, and lifestyles correlate with the choice of a future profession [50,51]. In other words, there is usually a higher impact on risk factors among young university students from degrees within the area of health, such as nutrition, dietetics, physical education, nursing and medicine, where physical appearance and concern for health are very important [22,52].

Bo et al. [50] pointed out that the students enrolled on health sciences courses such nutrition showed a high prevalence of EDs (specifically, one fifth), and those with pre-existing pathological eating patterns were especially inclined to enroll on health sciences courses. A European survey reported that 12.8% of students of nutrition presented an earlier or a current ED, such as anorexia nervosa, bulimia nervosa, and binge-eating disorder [53]. Along these same lines, Rocks et al. [54] and Toral et al. [33] found that the students of nutrition showed a double prevalence of the psychological characteristics and behavior often associated with EDs in comparison with students from other degrees.

Other studies, such as the one by Peña Salgado et al. [21] showed that student susceptibility to eating disorders was higher among the students enrolled on business administration, followed by law. In turn, Cancela and Ayán [55] found that physical inactivity and eating disorders had a significant prevalence among the students enrolled on primary school teaching and nursing. 

In relation to students of primary school teaching, some works showed that they have distorted attitudes and knowledge of the etiology of obesity, balanced nutrition and dietary regimes, and that the women especially presented risk factors such as inappropriate weight control techniques (use of laxatives and vomiting) [34,56].

Despite the abundance of the literature, some authors [27,34,57] point out the importance of to carry out more research to better understand the risk of suffering DEs in the university population. As noted out by [30] eating disorders are more prevalent among university students compared to the general population. In this regard,, the objective of this investigation will be centered on determining whether, in Spanish university students, variables such as gender, age, course, educational faculty, and BMI are associated with a higher risk of suffering from eating disorders (ED).

## 2. Materials and Methods

A transversal, descriptive survey of a probabilistic sample population was designed [58]. Different data sets were then compiled for the study: the sample characteristics, the instrument, the procedure, and the analysis of the results. 

### 2.1. Participants

The investigation took place at the University of Burgos (Spain). The sample was formed of 561 students taken from a population of 6,277 students. Stratified sampling by degree and faculty resulted in the selection of the participants from among the 26 degrees taught at the nine faculties of the university. The sample was calculated using the formula for finite stratified samples, at a confidence level of 95.6% and with an error margin of +/−4% [59]. Convenience sampling was used to group the individual respondents to the survey.

In all, 42.8% of the sample were men (*n* = 240) and 57.2% were women (*n* = 276), with an average age of 21.7 (*SD* = 4.1). Regarding their origin, 75% of the students were from Burgos and the province, 12% from the other Spanish provinces and 13% were from other countries (China, Mexico, and France). In relation to the course in which the students were enrolled, 37.1% were enrolled in the fourth year of studies, 28.5% in the second year, 21.7% in the third year, and 12.7% in the first year. Frequencies and percentages of students by educational faculty are shown in Table 1.

The 561 male and female students had average heights of 1.78 meters (*SD* = 0.06) and 1.64 meters (*SD* = 0.06), respectively. Their average actual weights were 74.51 kilos (*SD* = 9.98) and 58.61 kilos (*SD* = 8.81), respectively. The ideal weight of the male students was 73.29 kilos (*SD* = 7.80) (1.22 kilos less than their average actual weight), and the ideal weight of the female students was 55.11 kilos (*SD* = 6.25). The body mass indexes (BMI) of the men and the women were 23.28 (*SD* = 2.79) and 21.77 (*SD* = 2.94), respectively. In relation to the data on the BMI classification, 22 (3.9%) students were underweight, 451 (80.45%) were normal weight, 86 (15.3%) were overweight (>22 and ≤25) and 2 (0.4%) were obese.

### 2.2. Instrument

Two instruments were designed: one, ad hoc, to collect sociodemographic data (gender, age, residence, educational faculty, degree, and course enrolment), and another standardized instrument, specifically, the Spanish version of the Eating Disorder Inventory-Reference criterion (EDI-3-RF) developed by Garner [11].

The EDI-3-RF consists of a brief self-administered questionnaire on the risk of developing an ED, based on concerns over food and feeding, body weight, stature, and the presence of extreme behaviors to control weight [11]. The inventory is formed of three scales of risk:

1. Obsession with thinness (OT): a scale with 7 items that measure obsession with thinness, worry over food, and an intense fear of gaining weight. This scale is a good predictor of the appearance of binge eating and the development of an ED. The range of direct scores is from 0 to 20, where 12 is the critical value (situation of real risk of an ED) [34].

2. Bulimia (B): an 8-item scale used to evaluate problematic eating patterns (binges) as a response to negative emotional states, constituting a risk factor. The range of direct scores runs from 0 to 32, and the critical values are between 5 and 8, in accordance with the body mass index (BMI) of each individual.

3. Body Dissatisfaction (BD): includes 10 items that evaluate dissatisfaction with the general shape of the body and with the size of specific parts that that cause extraordinary concern among people with EDs (e.g., stomach, hips, thighs, and buttocks). The direct scores of the scale from 0 to 40 are on a qualitative range: 0–6 low, 7–27 medium, 28–40 high body dissatisfaction.

In addition, the instrument includes socio-demographic questions, individual weight records, and five behavioral questions that examine the presence of extreme behaviors to control weight. 

In particular, they are: (1) Presence of binge eating: range of direct scores between 0–5 where, the critical score is between 2 and 5; (2) Induced vomiting or purges: range of direct scores between 0–5, where the critical score is between 1 and 5; (3) Use of laxatives: range of direct scores between 0–5, where the critical score is between 1 and 5; (4) Physical exercise as a means of losing or controlling weight: range of direct scores between 0–5, where the critical score is 5; (5) Weight loss of nine kilos or more during the last six months: a dichotomous Yes/No question where the critical score is Yes [11]. The responses, grouped into two categories to facilitate the analysis, were as follows: No Risk (when the response to each one of the five questions was outside the criteria of pathology) and Risk (when the responses to each of these five questions indicated a risk of suffering an ED).

The psychometric data yielded values between 0.82 and 0.96 for internal consistency (Cronbach’s alpha) with the Spanish adaptation in Spanish clinical samples and between 0.64 and 0.92 in non-clinical samples [60]. Equally, García et al. [34] analyzed the internal consistency with Cronbach’s alpha, which yielded a value of 0.91, a reliability level that was considered excellent [61]. The value of internal consistency in the sample from the present study was 0.886 (Cronbach’s alpha).

### 2.3. Procedure

Before the study commenced, the authorization of the Bioethics Committee of the University of Burgos and informed consent forms from all participants were obtained. The questionnaire was administered by researchers with previous training, the application of which was possible thanks to the disinterested collaboration of teachers from the different faculties at which the 26 degree courses were taught. The class selection criteria and therefore the selection of the students to be administered the questionnaires was random. One of the researchers visited the classrooms, gave information on data confidentiality, and requested the informed consent of the participants. Questionnaire administration time fluctuated between 15 and 30 min.

### 2.4. Data Analysis

The data were processed with the statistical program SPSS (version 28). A descriptive analysis of the variables under study was performed using frequency tables and percentages. Likewise, contingency tables were used to observe the relations between categoric variables with the Chi-squared statistic, at a significance level of *p* < 0.05. The Student *t*-test was used for two independent samples (with Levene’s test for equality of variances) and a one-way ANOVA test with the post hoc Bonferroni test for more than two groups. The effect size was calculated for each of the significant differences (*d* of Cohen).

Gender, age, course, faculty and BMI constituted the independent variables. Two categories (man-woman) in relation to the variable gender were used for data collection. Three categories were used for the age variable: 20 years or less, 21-to-25 years, and 26 years or over. Four categories were used for the year of the course: 1. First year; 2. Second year; 3. Third year; 4. Fourth year. Nine categories were considered for the variable faculty (Table 1). Four categories were used for BMI: Underweight (≤18.5), Normal weight (>18 and ≤22), Overweight (>22 and ≤25) and Obesity (>25).

The results of the EDI-3-RF on the three scales of risk (Obsession with Thinness -DT-, Bulimia -B- and Body Dissatisfaction -BD-) and the five behavioral questions (binge eating, vomiting, laxatives, physical exercise, loss of weight), constituted the dependent variables.

Pearson correlation and simple linear regression analysis were used to analyze the relationship between the variables age and BMI in its quantitative version. 

## 3. Results

The results were drawn from the impact of the sociodemographic, academic and BMI as independent variables (IV) on the dependent variables (DV) previously described for the study.

### 3.1. Differences as a Function of Gender

With regard to the values of the central tendency among women and the dispersion of the scores on the three scales of risk, the mean average both for Obsession with Thinness (OT) (*M_women_* = 7.25 and *SD_women_* = 5.63; *M_men_* = 4.72 and *SD_men_* = 4.40) and for Body Dissatisfaction (BD) (*M_women_* = 11.61 and *SD_women_* = 8.11; *M_men_* = 7.83 and *DT_men_* = 6.61) were, in both cases, higher and significantly different among women (*p* < 0.000). An effect size was obtained (Cohen d index) considered as moderate (0.500 and 0.510, respectively) as it was located between 0.50 ≤ *d* ≤ 0.79. So, in the present study there is a rather small risk of random chance-based differences between both groups [62]. On the Bulimia (B) scale, the two measures (men and women) were similar (*M_women_* = 4.35 and *SD_women_* = 4.25; *M_men_*= 4.32 and *SD_men_* = 4.16, *p* = 0.938).

Contingency Tables were applied to examine the qualitative range of the Body Dissatisfaction (BD) scale and to analyze its relationship with gender (Table 2). A chi-square test revealed that there is a statistically significant relationship between gender and the qualitative range of the BD, χ^2^ (2) = 18.46, *p* < 0.001. The corrected residue value reveals that significantly more men than women presented low Body Dissatisfaction (z = 4.2). Likewise, more women than men presented moderate Body Dissatisfaction (z = 3.8).

In relation to the presence of risk factors -binge eating, vomiting, use of laxatives, physical exercise, and weight loss-, different chi-square tests revealed that there is only a statistically significant relationship between gender and the presence of risk for excess physical exercise, χ^2^ (1) = 8.79, *p* = 0.003. The corrected residual value determined that significantly more men than women are at risk for excess physical exercise as a way to control their weight (z = 3) (Table 3).

### 3.2. Differences as a Function of Age

Table 4 describes the mean scores and standard deviation obtained by the students according to their age on the OT, B and BD scales. An ANOVA test revealed that there was a significant difference in the scores obtained by the students only in the Bulimia scale, *F*_(2,558)_ = 4.52, *p* = 0.011. No significant differences were observed on the OT and BD scales as a function of age. 

The analysis of multiple comparisons after the Bonferroni statistical test was looking for significative relations between scale B and the groups at extreme ends of the age scale; the results for the students under 20 years old and over 26 years old were in favor of the first group (*p* < 0.010), in other words, in favor of the younger students. The effect size can be considered as moderate (*d* = 0.584). 

No significant differences were observed for the presence of binge-eating, vomiting, use of laxatives, physical exercise, and weight loss as a function of age. Nevertheless, the age range with higher percentages for all types of behavior was between 20 and 25 years old, except for the behavior of binge-eating where the age range extended from under 20 years old to people over 26 years old.

### 3.3. Differences as a Function of the Year of the Course

Table 5 shows the mean scores and standard deviation obtained by the students on the OT, B and DT scales, according to the year of the course in which they are enrolled. An ANOVA test established that there were significant differences in the scores obtained by the students only in the Body Dissatisfaction (BD) scale, *F*_(3,557)_ = 3.461, *p* = 0.016.

The multiple comparison analysis following the application of the Bonferroni statistic pointed to significative relations between the results of the BD scale for the 2nd and the 3rd year students, in favor of the 2nd year students (*p* = 0.029), with a small effect size (*d* = 0.338). These students therefore presented significatively higher levels of body dissatisfaction.

No significant differences were observed, as a function of the year, on the other two scales, DT and B, nor for the five behavioral symptoms (binge eating, vomiting, laxatives, physical exercise, and weight loss).

### 3.4. Differences as a Function of the Educational Faculty 

Table 6 shows the mean scores and standard deviations obtained by the students on the OT, B, and BD scales according to the faculty in which they are enrolled. An ANOVA test established that there were significant differences in the scores obtained by the students only in the B scale, *F*_(8,552)_ = 2.903, *p* = 0.004. 

A Bonferroni post-hoc test revealed that the differences are statistically significant between students from the Faculty of Science and students from the Faculties of Law (*p* = 0.021) and Health (*p* = 0.013), in favor of the latter two. With a small effect size (*d* = 0.070).

No significant differences were observed, in accordance with the faculty for the other two scales, DT and BD.

Significant differences were only found between the five behavioral symptoms for the presence of binge eating (*p* < 0.011), in relation to the Health faculty with 40.4% of students, followed by the Law faculty with 38.8% of students (a small effect size, *d* = 0.075). No other significant differences were observed between the other groups.

### 3.5. Differences as a Function of Body Mass Indexes (BMI)

Contingency Tables were applied to examine the BMI classification and to analyze its relation with gender (Table 7). 

A chi-square test reveals that there is a statistically significant relationship between gender and body mass index, χ^2^ (3) = 15.480, *p* = 0.001. The corrected residual value reveals that significantly more females than men are underweight (z = 2.8). Similarly, more men than females are overweight (z = 2.4).

Table 8 shows the mean scores and standard deviations obtained by the students on the OT, B, and BD scales according to the BMI classification. An ANOVA test established that there were significant differences in the scores obtained by the students in the OT scale, *F*_(3,557)_ = 6.487, *p* < 0.001, and in the BD scale *F*_(3,557)_ = 12.740, *p* < 0.001.

A Bonferroni post-hoc test revealed that in the OT scale, normal weight students scored significantly higher in thinness obsession than underweight students (*p* = 0.033). Similarly, overweight students scored significantly higher on thinness obsession than underweight students (*p* < 0.001) and normal weight students (*p* = 0.017). The effect size was small (*d* = 0.034). On the BD scale, it was found that overweight students scored significantly higher on body dissatisfaction than underweight students (*p* < 0.001) and normal weight students (*p* < 0.001). On the other hand, obese students scored significantly higher on body dissatisfaction than underweight students (*p* = 0.04) and normal weight students (*p* = 0.029). The effect size was small (*d* = 0.064).

In relation to the presence of risk factors -binge eating, vomiting, use of laxatives, physical exercise, and weight loss-, different chi-square tests revealed that there is only a statistically significant relationship between BMI classification and the presence of risk for loss of weight, χ^2^ (3) = 8.388, *p* = 0.039 (Table 9). 

The corrected residual value determined that significantly more overweight students than expected have lost 9 kilograms or more during last 6 months (z = 2.8). 

Table 10 presents the percentages of students as a function of age in three categories and BMI. In relation to the percentages of BMI in four ranges, it can be noted that the highest percentages based on their BMI are in Normal weight and Overweight for students from 21 to 25 years old (54.5% and 51.2%, respectively). In Underweight and Obesity equal percentage (50% and 50%) for students 20 years old or less, and 21 to 25 years old. A chi-square test revealed that there was no statistically significant relationship between age in ranges and BMI classification, χ^2^ (6) = 3.281, *p* = 0.773.

When analyzing these two variables quantitatively (*M_age_* = 21.72, *SD_age_* = 4.11; *M_BMI_* = 22.42, *SD_BMI_* = 2.97), a Pearson correlation revealed a positive relationship between the two variables that was statistically significant (*p* = 0.004) although with a low strength (*r* = 0.120). This may suggest that the older the age, the higher the BMI. 

In addition, a simple linear regression analysis indicated that age explains 1.3% of the variance of BMI. That is, in 1.3% of the cases, age predicts BMI [*F*_(1,559)_ = 8.234, *p* = 0.004; *R*^2^ = 0.015; *R*^2^ corrected = 0.013; Age in years (β = 0.120, *p* = 0.004)].

## 4. Discussion

The purpose of this investigation was to estimate whether, in Spanish university students, variables such as gender, age, course, educational faculty, and BMI are associated with a higher risk of suffering from eating disorders (ED), with the aim of improving the understanding of the risk of suffering from eating disorders in the university population

The results as a function of gender showed that, although women scored higher than men on the three scales of risk, the significative differences were found for Obsession with Thinness (OT) and Body Dissatisfaction (BD). These results and those of other works [5,7,10,18,21,62] are coincident, thereby supporting the theory that the female population is subjected to greater social pressure. Equally remarkable is that no significative differences were found between men and women for bulimic behavior (scale B). As is the case in the study by Kowalkowsk and Poínos [29], who found that emotional eating and uncontrolled eating are positively correlated in both sexes. This finding may be related with the results obtained by García et al. [34], who reported significantly higher scores for bulimic behavior among men. The authors proposed no explanation for that result, which they qualified as unexpected, although they pointed to a significant increase in BD and Eating Disorders (ED) among men from Western countries [41,42].

Moreover, significant differences were only found for practicing physical exercise among the five behavioral symptoms. In particular, men presented risks of excessive physical exercise as a form of controlling their weight. This notable point that Bo et al. [50] and González-Carrascosa et al. [7] also underlined is that sport is among the compensatory behaviors used by men suffering from some sort of ET, especially anorexia nervosa [63]. In the same line, Chin et al. [10] points out in a study among university students in the United Arab Emirates, that women who wanted to lose weight preferred dieting to physical exercise. While men preferred exercise to diet.

With regard to the differences as a function of age, the students under 20 years old presented a more acute problematic (bulimic) behavior than the older students. These students did not present a higher BMI than the rest of the age ranges. Specifically, 78.9% of the students 20 years old or younger were at a BMI of normal weight. Therefore, these results can be attributed to the critical age for the development of ED [3,5] and not to having a higher BMI than the rest. These results were similar to those of Sáenz et al. [6] who observed a greater risk of ED among university students under 19 years old. 

Although there were no significative differences in the presence of binge eating, vomiting, use of laxatives, physical exercise, and weight loss, the highest percentages for age were between 20 and 25 years, except for the behavior of binge eating where it was extended to 26 years old or over. Toro conducted a study in 2000 on people in need of specialized assistance, estimating that 4.5% of the population between 12 and 25 years fell into that category, alerting the health authorities to the susceptibility of people within that age range. García et al. [34] also found high risks among over 25-year-old students, suggesting the need to implement preventive intervention strategies that covered broader age groups than the standard ones. Equally, Cooper and Goodyer [64], in an assessment of concern over weight and body image among girls of different ages, concluded that despite the concerns over body form and appearance that arise at the start of adolescence, the behaviors relating to eating disorders occurred much later [20].

As a function of the year in which the student was enrolled, it was observed that the students in the second year presented significantly higher body dissatisfaction than those in the third year. Students are normally between 18 and 20 years old, in the second year, and students younger than 20 years old felt greater dissatisfaction with their bodies, which may possibly be related to that growth phase of life in late adolescence [7,18,21]. On the other hand, body dissatisfaction was accompanied by negative emotions that might be responsible for a bulimic type of ED among the students under 20 years old observed in this work and that adds to the risk of suffering an ED. These results are close to those of Gropper et al. [44], who pointed out that the first years of university are a critical period for the development of an ED [65]. Increased independence and responsibilities, as well as concern over their own identity all contributed in turn to the development of eating pathologies [1].

Differences were found as a function of the study faculty between the students enrolled at the Health Sciences Faculty (Occupational Therapy and Nursing Degrees) and at the Law Faculty (Law; Political Science and Public Management Degrees; double Degree in Law and Administration and Business Management). In particular, they showed a greater tendency to think of uncontrolled attacks of binge eating and a higher tendency to indulge in them (scale B).

From among the five behavioral questions, the students from these two faculties only showed a greater presence of binge eating.

These results reinforce the results found in the literature [18,33], in so far as a higher risk of ED was attributed to the students from Health Sciences, as a consequence of greater concern for health and physical appearance. It would be necessary to investigate whether the students in this study presented pathological EDs prior to their entry into university, which might moreover be a reason for their choice of this area of study [50,52].

The results referring to the students from the Law Faculty were congruent with those obtained by other authors such as Peña et al. [21] who found a higher percentage of students at risk of ED following the Business Administration and Law degrees. Perhaps the students of those degrees considered that they were under greater pressure than on other degree courses, due to esthetic ideals of beauty that have traditionally been attributed to students on degrees such as Law and Administration and Business Management. Nevertheless, investigation will have to continue to analyze the underlying causes of these results. Regarding the differences according to the BMI value, we found that women were underweight and men were overweight. This finding is in accordance with the scientific literature. For example, Radwan et al. [66] found that women desired a thinner or smaller body, as opposed to men who desire a more muscular body and a larger body size [10]. Similarly, authors such as Momeni et al. [5] and Alipour et al. [67] note that thinness was the most desirable body image for female college students.

On the other hand, we found that overweight students scored significantly higher in obsession with thinness (DT) and body dissatisfaction (BD) than their normal and underweight peers. Similarly, obese students have higher body dissatisfaction (BD) than their normal and underweight peers. These results in overweight and obese students are to be expected and are in line with the scientific literature [5,10,11]. These students suffer constant internal and external pressures to lose weight for health or social reasons. Numerous studies have found a positive correlation between body dissatisfaction and obsession with thinness with weight and BMI [8,10,11,29,30]. It is rare to find overweight subjects with low body dissatisfaction scores, although acceptance of body size without the need to lose weight is one of the primary goals of non-weight loss treatments for obesity [11,29,30].

With respect to students with normal weight showed greater obsession with thinness (DT) than those with low weight, data that seem particularly relevant. As reported by other researchers [5,11,68,69], if a person’s weight is within the normal range, high scores in thinness obsession indicate the possible presence of disturbing symptomatology. It should be noted that the same level of psychological distress (intense desire to be thinner) may have different meanings depending on weight [11]. For example, many patients with bulimia nervosa have a normal weight, but this value may represent a very significant loss of their previous weight [11]. However, as Momeni et al. [5] point out this negative evaluation of body image and weight is quite common among women in modern societies, even among those with a normal BMI.

## 5. Conclusions

In conclusion, the female university students from the University of Burgos presented as significantly underweight, and with higher levels of obsession with thinness and greater body dissatisfaction than the men in the same sample. In turn, this body dissatisfaction was significantly more present among students enrolled in the second year of the degree. The men, in addition, presented as significantly overweight, practiced significantly more physical exercise as a form of controlling their weight and the younger students under 20 years old and those from the Faculties of Health Sciences and Law presented significantly higher bulimic and binge-eating behaviors. In general, overweight students in the study score significantly high in obsession with thinness and body dissatisfaction, and have also lost 9 kilos or more during the last 9 months. Similarly, students with obesity have high body dissatisfaction. On the other hand, students with normal weight score significantly high in obsession with thinness, which may indicate an alarm factor.

The data obtained in this study, as well in other previous ones [10,23,24,70,71,72] reinforce the hypothesis that the female gender, at an age within the limits of early adolescence, the first year of the degree courses, certain university qualifications and a high BMI constituted factors that can influence the appearance or the continuance of risk-related patterns of eating, which could provoke one among various sorts of ED. In consequence, these results determine the need to implement measures that are specifically adapted to university students that stimulate heathy eating habits, improving the perception of their body image and reducing obsessive concerns over thinness. As García et al. [34] and Du et al. [30] pointed out, although maximum risk is reached during adolescence, the levels observed among university students are sufficiently important to propose these sorts of interventions. In addition, these symptoms are relatively stable during the university period [68,73].

In this sense, and with the aim of preventing ED among students at the University of Burgos (Spain), a group prevention program has been implemented this academic year (2022–2023).

Fortunately, recent systematic revisions and meta-analyses have demonstrated theoretical and methodological advances in the field of the prevention of EDs. The calculations of the latest meta-analyses suggest that at present over half (51%) of preventive interventions reduce the risk factors and somewhat over a quarter (29%) reduce the prevalence and the incidence of present and future eating pathologies [3,27,57,74,75]. Preventive eating disorder interventions may also be effective in preventing the onset of subclinical and full syndrome eating disorders [27]. Consequently, as Harrer et al. [27] and Du et al. [30] points out future research should investigate how such interventions can be effectively implemented in university settings, and how to motivate students to participate. Therefore, universities should consider implementing screenings for maladaptive eating behaviors for students and providing interventions as needed [30].

Finally, the limitations of this research are principally related with the EDI-3-RF inventory, as a self-administered questionnaire in which some of the responses could have been both false positive and false negative. Other limitations of the study are related to the lack of follow-up to assess whether the results are maintained over time. On the other hand, this is a cross-sectional study, so a single measurement of the study variables was collected. Consequently, it is not possible to infer causal relationships between the variables. Furthermore, since the data were collected from a sample of students from a medium-sized public university, the results cannot be generalized to the rest of the Spanish population. 

With regard to future investigations, important areas include the development of ED-related awareness-raising measures, especially within the context of Burgos University, and the evaluation of their benefits. Future research projects will evaluate the benefits of applying the ED prevention program, implemented in the context of the University of Burgos. Likewise, as future research, we will analyze the risk of suffering ED in university students in relation to other aspects of mental health, such as anxiety and depression, aspects on which we are currently working.

## Figures and Tables

**Table 1:** Frequency and percentage of students by academic year and educational faculty.

Educational Faculty and Degrees	Frequency	Valid Percentage
Sciences (degrees in Chemistry, and Food Science and Technology)	42	7.5
Law (degrees in Law, Law and Business Administration and Management, and Political Science and Public Management)	67	11.9
Economics (degrees in Business Administration and Management, degree in Finance and Accounting, and degree in Tourism)	67	11.9
Education (degrees in Early Childhood Education Teacher, Primary Education Teacher, Social Education, and Pedagogy)	120	21.4
HPS_Milanera (degrees in Technical Architecture, Agri-Food Engineering and the Rural Environment, Civil Engineering, and Road Technology Engineering)	38	6.8
HPS_Rio vena (degrees in Industrial Organization Engineering, Automatic and Industrial Electronic Engineering, Computer Engineering, and degree in Mechanical Engineering)	110	19.6
Humanities and communication (degrees in Audiovisual Communication, Spanish: Language and Literature, and History and Heritage)	53	9.4
Labor relations (degree in Labor Relations and Human Resources)	17	3.0
Health (degree in Nursing -2010 plan-, degree in Nursing -2015/16 plan-, and degree in Occupational Therapy)	47	8.4
Total	561	100

Note. HPS = Higher Polytechnic School.

**Table 2:** Results of contingency tables as a function of gender for the qualitative range of the Body Dissatisfaction (BD) scale.

Range of the Body Dissatisfaction (BD) SCALE						Gender		
	*N*	% Total	*n*	Men		*n*	Women	
				%	Corrected Residue		%	Corrected Residue
Low (0–6)	217	38.7	117	48.8	4.2	100	31.2	−4.2
Moderate (7–27)	329	58.6	119	49.6	−3.8	210	65.4	3.8
High (28–40)	156	2.7	4	1.7	−1.3	11	3.4	1.3
Total	561	100	240			321		

**Table 3:** Percentages of students presenting pathological behavior due to excessive physical exercise.

During the Past 3 Months, How Frequently Have You Exercised for 60 min or More to Control Your Weight?					Gender			
	*N*	% Total	*n*	Men		*n*	Women	
				%	Corrected Residue		%	Corrected Residue
No risk	541	96.4	225	93.8	−3	316	98.4	3
Risk	20	3.6	15	6.3	3	5	1.6	−3
Total	561	100	240			321		

**Table 4:** Descriptive statistics of the DT, B, and BD scales as a function of ageand ANOVA test.

BDI Scales Age Three Categories		*n*	*M*	*SD*	Standard Error	ANOVA				
							Quadratic MEAN	Degrees of Freedom	*F*	*p*
Obsession with Thinness (OT)	20 years old or less	218	6.65	5.455	0.369	Between groups	43.328	2	1.551	0.213
	21 to 25 years old	302	5.82	5.182	0.298	Within groups	27.942	558		
	26 years old or more	41	6.12	5.129	0.801	Total		560		
	Total	561	6.17	5.291	0.223					
Bulimia (B)	20 years old or less	218	4.77	4.585	0.311	Between groups	79.350	2	4.522	0.011
	21 to 25 years old	302	4.25	4.087	0.235	Within groups	17.550	558		
	26 years old or more	41	2.66	2.254	0.352	Total		560		
	Total	561	4.34	4.215	0.178					
Body Dissatisfaction (BD)	20 years old or less	218	10.61	8.068	0.546	Between groups	140.969	2	2.369	0.095
	21 to 25 years old	302	9.84	7.414	0.427	Within groups	59.502	558		
	26 years old or more	41	7.83	7.956	1.242	Total		560		
	Total	561	9.99	7.733	0.326					

Note. *M* = Mean; *SD* = Standard Deviation.

**Table 5:** Descriptive statistics of the DT, B, and BD scales as a function of year and ANOVA test.

BDI Scales Year Course		*n*	*M*	*SD*	Standard Error			ANOVA		
							Quadratic Mean	Degrees Freedom	*F*	*p*
Obsession with Thinness (OT)	1	71	7.07	5.773	0.685	Between groups	49.247	3	1.766	0.153
	2	160	6.59	5.355	0.423	Within groups	27.882	557		
	3	122	5.55	4.994	0.452	Total		560		
	4	208	5.90	5.211	0.361					
	Total	561	6.17	5.291	0.223					
Bulimia (B)	1	71	5.45	4.840	0.574	Between groups	44.105	3	2.502	0.059
	2	160	4.53	4.586	0.363	Within groups	17.628	557		
	3	122	3.89	3.893	0.352	Total		560		
	4	208	4.07	3.808	0.264					
	Total	561	4.34	4.215	0.178					
Body Dissatisfaction (BD)	1	71	9.28	7.663	0.909	Between groups	204.237	3	3.461	0.016
	2	160	11.61	8.109	0.641	Within groups	59.015	557		
	3	122	9.00	7.283	0.659	Total		560		
	4	208	9.57	7.578	0.525					
	Total	561	9.99	7.733	0.326					

**Table 6:** Descriptive statistics of the DT, B, and BD scales as a function of faculties and ANOVA test.

BDI Scales Faculties		*n*	*M*	*SD*		ANOVA			
						Quadratic Mean	Degrees Freedom	*F*	*p*
Obsession Thinness (OT)	Sciences	42	5.45	4.586	Between groups	21.587	8	0.769	0.631
	Law	67	6.66	6.134	Within groups	28.090	552		
	Economics	67	7.13	5.967	Total		560		
	Education	120	5.78	4.667					
	HPS_Milanera	60	5.57	5.347					
	HPS_Rio vena	88	5.83	4.962					
	Humanities-communication	53	6.36	5.335					
	Labor relations	17	6.35	4.358					
	Health	47	6.83	5.906					
	Total	561	6.17	5.291					
Bulimia (B)	Sciences	42	2.74	8.755	Between groups	50.221	8	2.903	0.004
	Law	67	5.57	8.123	Within groups	17.300	552		
	Economics	67	4.61	7.121	Total		560		
	Education	120	3.80	8.327					
	HPS_Milanera	60	3.75	7.074					
	HPS_Rio vena	88	4.09	7.226					
	Humanities-communication	53	4.72	7.429					
	Labor relations	17	3.94	8.041					
	Health	47	5.91	7.442					
	Total	561	4.34	7.733					
Body Dissatisfaction (BD)	Sciences	42	10.02	2.231	Between groups	55.073	8	0.920	0.499
	Law	67	10.01	4.778	Within groups	59.861	552		
	Economics	67	10.57	3.992	Total		560		
	Education	120	10.48	4.012					
	HPS_Milanera	60	7.70	3.722					
	HPS_Rio vena	88	9.80	4.712					
	Humanities-communication	53	9.96	4.276					
	Labor relations	17	11.82	2.436					
	Health	47	10.53	4.840					
	Total	561	9.99	4.215					

**Table 7:** Results of contingency tables as a function of gender for the BMI classification.

BMI Classification	Gender						Total	
	Men			Women				
	*n*	%	Corrected Residue	*n*	%	Corrected Residue	*N*	%
Underweight	3	1.3%	−2.8	19	5.9%	2.8	22	3.9%
Normal weight	188	78.3%	−1.1	263	81.9%	1.1	451	80.4%
Overweight	47	19.6%	2.4	39	12.1%	−2.4	86	15.3%
Obesity	2	0.8%	1.6	0	0.0%	−1.6	2	0.4%
Total	240	100.0%		321	100.0%		561	100.0%

**Table 8:** Descriptive statistics of the DT, B, and BD scales as a BMI classification and ANOVA test.

BDI Scales BMI Classification		*n*	*M*	*SD*		ANOVA			
						Quadratic Mean	Degrees Freedom	*F*	*p*
Obsession Thinness (OT)	Underweight	22	2.82	3.459	Between groups	176.422	3	6.487	0.001
	Normal weight	451	6.00	5.331	Within groups	27.197	557		
	Overweight	86	7.84	4.958	Total		560		
	Obesity	2	10.00	4.243					
	Total	561	6.17	5.291					
Bulimia (B)	Underweight	22	2.55	2.890	Between groups	29.721	3	1.679	0.171
	Normal weight	451	4.46	4.227	Within groups	17.706	557		
	Overweight	86	4.22	4.399	Total		560		
	Obesity	2	2.00	1.414					
	Total	561	4.34	4.215					
Body Dissatisfaction (BD)	Underweight	22	5.59	5.279	Between groups	716.692	3	12.740	0.001
	Normal weight	451	9.44	7.537	Within groups	56.255	557		
	Overweight	86	13.67	7.724	Total		560		
	Obesity	2	24.50	10.607					
	Total	561	9.99	7.733					

**Table 9:** Percentages of students presenting pathological behavior due of loss of weight as a function of BMI classification.

During the Last 6 Months You Have LOST 9 Kg or More			BMI Classification											
			Underweight			Normal Weight			Overweight			Obesity		
	*N*	% Total	*n*	%	Corrected Residue	*n*	%	Corrected Residue	*n*	%	Corrected Residue	*n*	%	Corrected Residue
No risk	539	96.1	22	4.1	1	437	81.1	2	78	14.5	−2.8	2	0.4	0.3
Risk	22	3.9	0	0	−1	14	63.6	−2	8	36.4	2.8	0	0	−0.3
Total	561	100	22			451			86			2		

**Table 10:** Percentages of students as a function of age in three categories and BMI.

Age Three Categories			BMI Classification											
			Underweight			Normal Weight			Overweight			Obesity		
	*N*	% Total	*n*	%	Corrected Residue	*n*	%	Corrected Residue	*n*	%	Corrected Residue	*n*	%	Corrected Residue
20 years old or less	218	38.9	11	5	1.1	172	78.9	−0.7	34	15.6	0.1	1	0.5	0.3
21 to 25 years old	302	53.8	11	3.6	−0.4	246	81.5	0.7	44	14.6	−0.5	1	0.3	−0.1
26 years old or more	41	7.3	0	0	−1.3	33	85.5	0	8	19.5	0.8	0	0	−0.4
Total	561	100	22			451			86			2		

## Data Availability

The authors undertake to provide the data when requested.
